# Immunoregulation through IL-10 gene expression and the fate of cytotoxic T lymphocyte-mediated tumor immunotherapy

**DOI:** 10.4103/0971-6866.50862

**Published:** 2009

**Authors:** Nitya G. Chakraborty

**Affiliations:** Department of Medicine, University of Connecticut School of Medicine, Farmington, CT 006030-1628, USA

**Keywords:** Tumor antigens, Cytotoxic T Lymphocyte (CTL) Regulatory T cells (Tregs), Interleukin-10 (IL-10)

## Abstract

Gene analysis of tumor associated antigens revealed that tumor antigens are all normal gene product. Inducing tumor reactive cytotoxic T lymphocytes (CT) in the patients is same as inducing autoimmunity in the patients. Immunosuppressive cytokine interleukin-10 (IL-10) plays an important role in maintaining homeostasis or tolerance. To break the tumor tolerance, blocking and IL-10 secretion or intervention in the pathways of IL-10 gene activation is indeed important.

## Introduction

**Figure 1:**
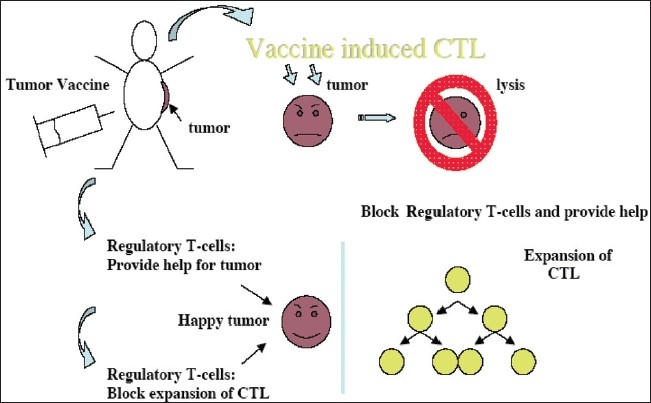
Vaccine induced CTL response

Vaccine therapy with tumor (cancer)-associated antigens or tumor-derived heat shock protein is a promising development in the field of tumor immunotherapy. The majority of the clinical trials were/are either aimed at the generation of a tumor-specific cytolytic T lymphocyte (CTL) response in the patients or injecting the patients with *ex vivo* expanded tumor antigen-specific CTL. Following vaccine therapy or CTL infusion, several studies have documented tumor-specific CTL in patients′ blood at the vaccine site and also at distant (regressing) tumor sites. Dramatic clinical antitumor responses have even been seen in advanced cancer following this type of immunotherapy. The major problem with this approach is that the *in vivo* expansion of antigen-specific CTL is neither persistent nor robust. The reason for nonexpansion of vaccine-induced CTL is better understood now. The following Figure 1 represents the overall picture of the *in vivo* reaction.

Genetic analysis of tumor-associated antigens reveals that although the antigens are tumor associated, they are all normal gene products or “self-antigens.” Tumor grows from and within the body and the immune system clearly tolerates the growth of metastatic tumors. Inducing a CTL response or injecting CTL against a tumor is, in effect, inducing an autoimmunity in the patients. Thus, pre-existing tumor-tolerating elements in the patient stops the expansion of vaccine-induced CTL or injected CTL. Two important elements involved in induction and maintenance of peripheral tolerance are known: (a) naturally occurring CD4+ CD25+ regulatory T cells or n-Tregs[1,2] And (b) induced CD4+ CD25+ regulatory T cells or i-Tregs. These are found to downregulate the CTL response by elaborating various suppressor cytokines such as interleukin (IL)-10 and transforming growth factor-β in various concentrations and combinations.[3,4] In human tumors, IL-10 was found to be the most important tumor.

## Innate and adaptive immunity and immune homeostasis

**Figure 2:**
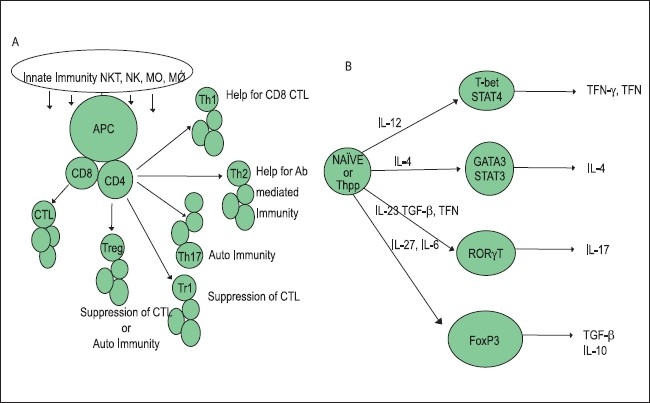
Innate and adaptive Immune response and the corresponding gene expressions

The innate immune system consists of a diverse collection of host defenses that include physical and chemical barriers and cellular components, like macrophages, neutrophils, and natural killer cells, all committed to provide an instant protective response via secretion of inflammatory cytokines and antibacterial peptides. The adaptive component of the immune system is sequentially activated by the activated innate system and is represented by CD4+ and CD8+ T lymphocytes and B lymphocytes. The exit of the T cells from the thymus to the peripheral tissues and their recirculation through the blood and the lymphatic organs makes them able to interact with the antigens presented by the antigen-presenting cells (APCs) in the context of HLA class I or class II molecules. Induction of an effective cytotoxic T cell response require two signals, namely engagement of T cell receptor (TCR) with the antigen (presented by APC in the context of HLA class I antigen) and engagement of the costimulatory molecules such as CD28 on CTL and B7.1 and 7.2 on APC. The process leads to a successful activation and clonal expansion of the antigen-specific CTLs. Helper T cells (CD4+ lymphocytes) participate by their engagement with the APC through the TCR in the context of class II antigens, leading to secretion of cytokines important in a cross-talk with both B cells and cytotoxic T cells that leads to an amplification of the immune response. While an effective immune response is important against an invading antigen, a robust and uncontrolled immune response could lead to severe and life-threatening tissue injury. Therefore, a critical balance needs to be achieved between a robust CTL and a T helper 1type response on one hand and its regulation on the other to protect against the injury caused by uncontrolled immunity. A regulatory activity thus generated limits the damage from overactivity of the immune system. This function is effectively performed by the suppressor-type T lymphocytes, now called as regulatory T cells, and is identified by the expression of a distinct CD4+ CD25+ FoxP3+ phenotype. Two types of regulatory T cells exist, naturally occurring T regs or n-Tregs(1, 2) and induced T regs, induced according to the need or i-Tregs.[3,4] The homeostasis is achieved by the regulatory T lymphocytes. Figure 2A and B describe the innate and adaptive responses and the corresponding gene expressions. Experiments in animal models and human systems have established that IL-10-producing Treg/suppressor cells play a major role in maintaining homeostasis by controlling the inflammatory immune response.[5–8] In the event of immune response against cancer induction of i-Treg, cells at the site of the cancerous growth prevent tumor antigen-specific CTL from their proper functioning and expansion.

Immunosuppression by IL-10 producing Treg or Tr1 type of cells. IL-10 is a cytokine that shows potent immunosuppressive and anti-inflammatory activity.[9] It has the ability to inhibit the production of pro-inflammatory cytokines, such as tumor necrosis factor-α, IL-1, IL-6, and IL-12 as well as IL-2 and interferon (IFN)-γ. The important role of IL-10 in controlling inflammation is exemplified by IL-10-/- mice, which develop inflammatory bowel disease.[10–16] Multiple studies have shown the roles of IL-10 in suppressing the immune response to infectious disease and suppressing the development of autoimmune disease. Altogether, the role of IL-10 appears to be to limit the effect of pro-inflammatory cells and the cytokines they produce to prevent damage to the host and to maintain self-tolerance.

Initially, IL-10 was described as a product of the Th2 type of cells. Subsequent work has shown that IL-10 is produced by multiple cell types, including Th1 cells, Tr1 cells, NKT cells, B cells, macrophages, keratinocytes, and carcinoma cell lines.[10–15] Despite the fact that various types of cells can produce IL-10, little is known about the control of IL-10 transcription and gene expression. It is known that IL-10 expression is controlled at both the transcriptional and the post transcriptional levels.[14–16] Two separate studies have shown that the only essential element of the mouse IL-10 promoter is a single Sp1/Sp3 site.[16] Because Sp1/Sp3 proteins are ubiquitous, the mechanism controlling IL-10 expression in different cell types is not clear. Moreover, it is not clear whether a single Sp1/Sp3 site in theIL-10 promoter is sufficient for driving high-level IL-10 expression in Th2 cells.

## Control of the immune response via circulating 5′ adenosine monophosphate (AMP) and the adenosine pathway

A subpopulation of peripheral blood lymphocytes (PBL) expresses CD73.[16] CD73/ecto-5nucleotidase (ecto-5-NT), is a 70-kD glycosyl-phosphatidylinositol-linked molecule that can be detected in several different tissues and cell types.[17] The natural ligand(s) of CD73 are currently not known. Enzymatic activity of ecto-5-NT catalyzes the extracellular dephosphorylation of nucleoside monophosphates to their corresponding nucleosides. This process enables the uptake of adenosine, inosine, and guanosine into the cell and their subsequent reconversion into ATP and GTP in the purine salvage pathway. The role of ecto-5-NT probably differs in various tissues. Naïve CD4+ cells differentiate to become various functional CD4, as shown in Figure 2. It has been shown that there are uncommitted prime precursors (Thpp) and Treg cells that express CD73. These Treg and Thpp cells also share other properties and can differentiate from naive CD4 T cells under similar conditions. Adenosine produced by either cell type suppresses proliferation of effector CD4 T cells (Th1, Th2, or Th17) and inhibits the production of cytokines. Thus, the CD73/adenosine pathway could be a potent suppressive pathway of Treg cells that confers a suppressive anti-inflammatory function. Infiltration of either n-Treg or i-Treg cells at inflammatory sites could potentially convert the 5′ AMP generated by CD39 expressed on neutrophils or dying cells into the anti-inflammatory mediator adenosine and, thus, dampening excessive immune reactions.[17–21] In any event for the expansion of tumor antigen-specific CTL response, blocking of the infiltration of Tregs at the tumor site is important. It appears if, by any means, those uncommitted precursors have the potentiality to become potent suppressors of the induced CTL. It has been shown that a chemotherapeutic agent fludarabine, used for the treatment of certain types of blood cancer, if used at a dose (100 µg) lower than the standard dose (25 mg) could reduce the number of regulatory T cells in the patients.[22,23] Fludarabine has a binding affinity for CD73 expressed by certain CD4+ cells and those are termed as uncommitted prime precursors with a potential to be effective suppressors of the inflammatory responses.[20]

## Presence of fludarabine in low dose in culture-maintained functional CTLs generated in cultures with total PBL

Several studies, preclinical or clinical, have shown that the CTL responses induced by vaccine therapy in patients or *in vitro* in stimulation cultures are short lived.[24–26] We have also shown in *in vitro* cultures that the induction of CD8+ CTL responses decline in mixed culture with total PBL whereas CTL responses in culture with purified CD8+ cells remain significantly longer in cultures.[25] Table 1 summarizes the results of *in vitro* cocultures to induce tumor antigen-specific CTL response using total PBL and purified CD8+ cells.

**Table 1:** Fate of tumor antigen specific CTL generated in cultures

CTL generated with				Functional analysis		
	Cytokine production			Cytolytic function		
		
	Day 7	Day 14	Day 21	Day 7	Day 14	Day 21
Total PBL	+	++	--	+-	+	--
CD8+ cells	+	++	+++	+-	++	++

We have studied the effect of the drug fludarabine at a very low dose (10 ng/ml) in cultures with PBL from patients and normal donors. Phenotypic and functional activity of the expanded CTL in cultures was analyzed.[26] The cultures were fed every other day with appropriate cytokine and with and without the drug and were restimulated with appropriate stimuli every 7–10 days. Culture conditions and cytokine doses are described in previously published articles.[25–27] The cytokine secretion and cytolytic activity assays with the responding CTLs from the cultures were carried out after 14 and 28 days from the initiation of the cultures. The statistical comparison of the data was performed within the different conditions of the cultures with PBL and these were then further compared with *in vitro* cocultures (IVCS) with CD8+ cells, as described.[27^-29^] The following [Tables 2 and 3] represent the data of IFN-γ response and cytotoxicity assays, respectively, with the expanded CTL populations. For cytokine assays, after 14 and 28 days, some of the cells were removed from the cultures and washed three times in phosphate-buffered saline to remove cell surface-bound cytokines. These cells were then counted and stimulated separately in 96-well plates (1 × 10^5^ cells/well) with T2 cells pulsed with Mart-1 peptide or a control peptide or with T2 cells alone (responder:stimulator, 10:1) as previously described.[25–28] After 24 h, the supernatants from the wells were collected and cytokine assays by enzyme-linked immunosorbent assay for IFN-γ and IL-10 were performed. Table 2 represents the data with PBL only and with PBL, where the drug fludarabine was present continuously. It is clear that the presence of fludarabine generated better antigen-specific cytokine-producing cells when compared with control PBL IVC. In some of the control PBL IVCs, the IFN-γ-producing ability was totally lost and the difference found using the different culture conditions was found to be very significant (*P* < 0.05). The expanded antigen-specific cells in the cultures with fludarabine continued to be functional for a longer period as compared with control PBL cultures in an autologous-DC-based restimulation system.[29] We have also observed [Table 3] a significantly elevated cytolytic activity of the responder cells from PBL IVC with fludarabine against both Mart-127-35 peptide-pulsed target T2 and against a Mart-1-positive HLA-A2+ tumor cell line PT-M (as target cells with naturally processed antigen). It was observed that the antigen-specific killing was HLA-A2 restricted and killing was blocked in the presence of anti-HLA-A2 antibody. The extent of the killing of the target cells was very significant at all the effector-to-target ratios used because the cytotoxicity assay was performed in the presence of a 50-fold excess of cold K-562 cells (*P* < 0.02).

**Table 2:** IFN-γ synthesis by responding cells from the co cultures with PBL in the presence and absence of Fludarabine

Cell from	IFN-γ (pg/ml) when re stimulated with		
	
	T2 alone	T2+MAGE-3_278-9_	T2+MART-I_27-35_
Control PBL IVC Day 7	25±8	20±10	115±35
Control PBL IVC Day 14	5±2	15±5	300±50
Control PBL IVC Day 28	10±5	15±4	20±5
PBL IVC in presence of FLU day 7	50±18	80±45	220±55
PBL IVC in presence of FLU day 14	0	0	330±75
PBL IVC in presence of FLU day 28	0	0	350±66

**Table 3:** Cytotoxicity assay with responder cells from IVC with total PBL after 28 days of initiation of the culture

Effector	Target cells	% lysis at E:T			
			
		40	20	10	5
Control PBL IVC	T2	0	0	0	0
	T2+ Mart 1 pep	5.6 ± 2.1	0	0	0
	T2+ Cone pep	0	0	0	0
	PT-M PT-M + anticlass I	0	0	0	0
	Ab	0	NT	NT	NT
	PT-M + anticlass II Ab	0	NT	NT	NT
PBL IVC+ fludarabine	T2	0	0	0	0
	T2+ Mart 1 pep	40.2 ± 10.1	30.6 ± 7.9	18.4 ± 5.1	6.9 ± 2.8
	T2+ Cone pep	2.0	0	0	0
	PT-M	26.6 ± 6.9	27.4 ± 7.8	17.4 ± 4.6	10.2 ± 4.6
	PT-M + anticlass I Ab	4.0	NT	NT	NT
	PT-M + anticlass II Ab	28.4 ± 11.3	NT	NT	NT

Cytokine IFN-γ synthesis by responder cells from the cocultures with total PBL with or without fludarabine (FLU) at different times after initiation of the cocultures.

## Conclusion

Animal model experiments have established the principles of immunotherapy for cancer, but in order to explore tolerance to tumor by the patients, each patient must be analyzed individually. Different patients having similar types of cancer might have different mechanisms for tolerating their cancer (number and type of cells, type and/or quantity of cytokines). While investigating tolerance to tumors, it is difficult, if not impossible, to extrapolate results from genetically identical animal models with experimental tumors to genetically diverse humans with spontaneously arising cancers. Hundreds of articles have been published so far on regulatory T cells and suppression of the immune response against tumors. Very few articles are published that talk about the blocking of the induction and expansion of regulatory T cells, especially inducible regulatory T cells. In this connection, the use of an already-approved drug, used at a suboptimum dose, along with vaccine therapy that could block induction and expansion of regulatory T cells and prolong the survivability of induced CTL, is very important.
